# *In vitro* bacterial vaginosis biofilm community manipulation using endolysin therapy

**DOI:** 10.1016/j.bioflm.2022.100101

**Published:** 2022-12-29

**Authors:** William Johnston, Alicia Ware, Willemijn Frederique Kuiters, Christopher Delaney, Jason Lee Brown, Suzanne Hagen, David Corcoran, Matthew Cummings, Gordon Ramage, Ryan Kean

**Affiliations:** a Department of Biological and Biomedical Sciences, Glasgow Caledonian University, Cowcaddens Road, Glasgow, G4 0BA, United Kingdom; b Glasgow Biofilm Research Network, United Kingdom; c Oral Sciences Research Group, University of Glasgow, 378 Sauchiehall Street, G2 3JZ, Glasgow, United Kingdom; d Midwifery and Allied Health Professions Research Unit, Glasgow Caledonian University, Cowcaddens Road, Glasgow, G4 0BA, United Kingdom; e CC Biotech Ltd, Imperial Translation and Innovation Hub, 84 Wood Lane, London, W12 0BZ, United Kingdom

**Keywords:** Biofilm, Bacterial vaginosis, Endolysin, Gardnerella vaginalis, Reproductive health

## Abstract

Bacterial vaginosis (BV) affects approximately 26% of women of childbearing age globally, presenting with 3–5 times increased risk of miscarriage and two-fold risk of pre-term birth*.* Antibiotics (metronidazole and clindamycin) are typically employed to treat BV; however the success rate is low due to the formation of recalcitrant polymicrobial biofilms. As a novel therapeutic, promising results have been obtained *in vitro* using *Gardnerella* endolysins, although to date their efficacy has only been demonstrated against simple biofilm models.

In this study, a four-species biofilm was developed consisting of *Gardnerella vaginalis, Fannyhessea vaginae, Prevotella bivia* and *Mobiluncus curtisii*. Biofilms were grown in NYC III broth and treated using antibiotics and an anti-*Gardnerella* endolysin (CCB7.1) for 24 h. Biofilm composition, viability and structure were assessed using colony counts, live/dead qPCR and scanning electron microscopy.

All species colonised biofilms to varying degrees, with *G. vaginalis* being the most abundant. Biofilm composition remained largely unchanged when challenged with escalated concentrations of conventional antibiotics. A *Gardnerella-*targeted endolysin candidate (CCB7.1) showed efficacy against several *Gardnerella* species planktonically, and significantly reduced viable *G. vaginalis* within polymicrobial biofilms at 1 to 4X pMIC (p < 0.05 vs. vehicle control).

Collectively, this study highlights the resilience of biofilm-embedded pathogens against the currently used antibiotics and provides a polymicrobial model that allows for more effective pre-clinical screening of BV therapies. The *Gardnerella*-specific endolysin CCB7.1 demonstrated significant activity against *G. vaginalis* within polymicrobial biofilms, altering the overall community dynamic and composition.

## 1 Introduction

Bacterial vaginosis (BV) affects approximately 26% of women of childbearing age globally [1,2], and has a profound psychological impact on patients with significant consequences for prenatal health. The condition is characterised by compositional alterations in the vaginal microbiome, resulting in a shift from a lactobacilli-dominated microbiome to one composed largely of BV-associated anaerobes - including *Gardnerella* spp. (*G. vaginalis, G. leopoldii, G. piotii, G. swidsinskii*)*, Fannyhessea vaginae, Prevotella bivia* and *Mobiluncus curtisii* [[3], [4], [5], [6]]. This microbial shift gives rise to associated symptoms including foul odour, burning during urination, vaginal discharge and itching. Furthermore, there are strong links between BV and risk of miscarriage, pre-term birth and sexually transmitted infections [7].

During the onset of BV, anaerobes such as *Gardnerella* spp. displace commensal lactobacilli to form biofilms on the vaginal epithelium [8]. This results in an elevated pH and colonisation of a diverse polymicrobial community, contributing to BV onset, progression and treatment failure [9]. Current treatments for BV are extremely limited and most cases are treated using antibiotic therapy (e.g. metronidazole and clindamycin). However, antibiotic regimens have an unacceptably large rate of relapse with >50% of patients suffering from BV recurrence within 6 months [10], believed to be driven, in-part, by the recalcitrance of these microbial biofilms [4]. As such, there is an unmet need to develop novel therapies for BV and evaluate their efficacy against biofilm-embedded bacteria.

One promising avenue in this regard is the use of bacteriophage-derived endolysin proteins which are produced during the lytic phase of phage replication, and act to degrade peptidoglycan from within bacterial cells to allow release of progeny [11]. Given this mechanism of action, these enzymes have good promise as therapeutics to be harnessed by clinicians. Previous studies have shown these enzymes are capable of lysing Gram-positive bacteria which lack an outer lipopolysaccharide membrane when added exogenously to culture media and more complex models of disease [[12], [13], [14]]. Interestingly, endolysins have also shown promise as antimicrobial agents against Gram-negative bacteria [15], despite their diderm cell wall structure. In addition, the targeted nature of bacteriophages and corresponding endolysin proteins mean they display high specificity for organisms, lending themselves to selective degradation of pathogens within complex microbial ecosystems without disruption of commensal flora [11]. Collectively, these features have resulted in the development of endolysins which target *Gardnerella* spp. – predominant members of BV-associated biofilms which contain a thin peptidoglycan layer [6,16].

Thus far, several endolysins directed against *Gardnerella* spp. have demonstrated good activity *in vitro*. For example, one endolysin (PM-477) was found to be highly selective for *Gardnerella* spp. [14], capable of eliminating mono-species *G. vaginalis* biofilms [17] and significantly reducing *G. vaginalis* load in dual-species biofilm models [18]. Likewise, recent screening of *Gardnerella* genome sequences has identified a library of endolysin candidates which prevent *G. vaginalis* biofilm formation and degrade pre-formed mono- and dual-species biofilms [12]. This library comprised 84 endolysin candidates which are diverse at the sequence and structural levels, with 3 different enzymatic active domains and 4 cell wall binding domains, with convergent bioactivities. Of these, several candidates were found to be particularly active against *G. vaginalis* mono-species biofilms, including CCB7.1 which comprises a C-terminal SH3b cell-wall binding domain and N-terminal LysA-like GH25 enzymatic active domain.

Whilst promising results have been obtained thus far, the screening of these proteins *in vitro* has been limited to planktonic organisms or simple (mono-, dual-species) biofilm models, which do not truly capture the complexity and diversity of the microenvironment in BV. As such, the current project aimed to build upon previous expansions in this field by developing a multi-species biofilm model that can be used for more robust high-throughput screening of novel compounds in BV treatment. The model consisted of four anaerobes frequently isolated in BV (*G. vaginalis*, *F. vaginae, P. bivia* and *M. curtisii*), with each species previously shown to exhibit monospecies biofilm formation [19]. Notably, both *F. vaginae* and *P. bivia* have been reported to influence *Gardnerella* biofilm-related gene expression *in vitro* [20], with the presence and persistence of *M. curtisii* associated with BV recurrence [5]. This biofilm model was initially assessed against current front-line antibiotics (metronidazole, clindamycin) and subsequently against a *Gardnerella* targeted phage endolysin candidate - CCB7.1 [12]. We sought to characterise the efficacy of this protein against our more complex polymicrobial system, lending further credence to the clinical utilisation of endolysin proteins.

## 2 Methods

### 2.1 Microbial culture and standardisation

The four species included in the core biofilm model were *Gardnerella vaginalis* (ATCC 14018), *Fannyhessea vaginae* (DSM 15829, formerly *Atopobium vaginae*), *Prevotella bivia* (DSM 20514) and *Mobiluncus curtisii* (CCUG 21018T). Additional *Gardnerella* isolates (*G. vaginalis* UG860107, *G. swidsinskii* CCUG 72429T and *G. piotii* CCUG 72427T) were also used for endolysin testing. All isolates were cultured onto Columbia agar (Merck Millipore, Cork, Ireland) containing 5% defibrinated horse blood (E&O laboratories, Bonnybridge, UK). For all isolates, agar plates were placed in the anaerobic cabinet overnight before culturing. Once grown, bacteria were stored on glycerol beads at −80 °C and re-cultured as required.

Liquid cultures were prepared by inoculating colonies into New York City III broth supplemented with 10% (v/v) inactivated horse serum (herein termed NYC III), which has been previously shown to be the optimal growth medium biofilm formation of BV-associated anaerobes [19]. Medium was incubated in the anaerobic cabinet 1 day prior to use. All bacterial culture was performed at 37 °C anaerobically within an anaerobic cabinet (Don Whitley Scientific MACS MG-500) with anaerobic gas influx (5% CO_2_, 10% H_2_ and 85% N_2_).

Following growth periods of 24 h (*Gardnerella* spp.) to 48–72 h (*F. vaginae, P. bivia, M. curtisii*), cultures were standardised to a desired cell concentration of 1 × 10^8^ CFU/mL for each organism using published standardisation optical density concentrations from target species as guidance which were independently confirmed internally using CFU/mL counting [[20], [21], [22]].

### 2.2 Endolysin production

Recombinant endolysins candidates were produced as previously reported [12]. In short, pCCB7.1, a pET30a(+) vector harbouring the codon optimised *ccb*7.1 gene sequence, was transformed into *E. coli* BL21(DE3). Transformants were grown in LB containing 25 μg/ml kanamycin (37 °C, 200 rpm) to OD_600_ 0.5–0.6 before induction of gene expression with 0.1 mM IPTG. Cultures were further incubated at 16 °C, 170 rpm for 12 h. Cells were recovered by centrifugation, (4000×*g*, 4 °C, for 20 min), and resuspended in chilled lysis buffer (PBS pH 7.4, 300 mM NaCl, DNase I (0.1 μg/mL) (New England Biolabs)) prior to sonication (amplitude 60%; 2 s on/off over 10 min for 2 cycles (Homogenizer UP100H, Hielscher)). Total soluble recombinant protein was obtained by centrifugation at 20,000×*g*, 4 °C, for 40 min.

CCB7.1 was purified to >95% homogeneity by immobilised metal affinity chromatography and size exclusion chromatography [12]. Total soluble protein was applied to capped PD-10 columns (GE Healthcare) containing Ni-NTA agarose (Qiagen) pre-equilibrated with lysis buffer. Capped columns were agitated at 4 °C for 1.5 h before being uncapped and washed with 10 column volumes (CV) of lysis buffer supplemented with 20 mM imidazole. Recombinant CCB7.1 was eluted from the PD-10 column via gravity flow using 1 CV of elution buffer (PBS pH 7.4, 300 mM NaCl, 200 mM imidazole) and stored on ice. Crude fractions containing CCB7.1 were dialysed into lysis buffer, pooled and concentrated using centrifugal filters. Concentrated CCB7.1 was further purified via SEC using a HiLoad 16/600 Superdex 75 pg column (GE Healthcare) preequilibrated with lysis buffer with a flow rates of 1 mL/min. Purified proteins were dialysed into buffer 1 (200 mM 2-(N-morpholino) ethanesulfonic acid (MES) pH 5.6, 150 mM NaCl, 4 mM dithiothreitol (DTT), 10% glycerol), for storage and continued use.

### 2.3 Drug preparation

Endolysin candidates were supplied by CC Biotech Ltd. in buffer 1. Antibiotics were purchased as powders (Sigma-Aldrich, Gillingham, UK) and prepared to stock concentrations of 5000 μg/mL and 10000 μg/mL in 100% ethanol and sterile distilled water for metronidazole and clindamycin, respectively. Stock solutions were freshly prepared for each set of experiments and aliquots stored at −20 °C between technical replicates. Working concentrations were prepared by dilution in NYC III medium (antibiotics) or buffer 1 where appropriate.

### 2.4 Planktonic MIC testing

Planktonic minimum inhibitory concentrations (pMICs) for antibiotics (clindamycin, metronidazole) and endolysins were performed using the CLSI M11-A8 broth microdilution method in NYC III, as reported previously for *G. vaginalis* [12,14]. The same experimental protocol was followed for *G. vaginalis* ATCC 14018, *G. vaginalis* UG860107, *G. swidsinkii* CCUG 72429T and *G. piotii* CCUG 72427T. In brief, bacteria were initially standardised to 1 × 10^8^ CFU/mL in NYC III, and then further diluted to 1 × 10^6^ CFU/mL in sterile medium (1:100). Antibiotics and endolysins were serially diluted 1:2 in sterile medium. Drugs were initially added at 100 μL to 96-well round bottom microtiter plates at double the required concentration, followed by 100 μL of 1 × 10^6^ CFU/mL *G. vaginalis*. The final bacterial concentration in wells was therefore 5 × 10^5^ CFU/mL as is recommended for pMIC testing [23]. Untreated controls (bacteria only) and negative controls (NYC III broth) were included in each assay run. Microtiter plates were incubated for 24 h anaerobically at 37 °C, with pMIC values determined by resuspending pellets and measuring well absorbance at OD_550_ using a microtiter plate reader (BMG labtech, Ortenberg, Germany). Results are presented as a percentage inhibition in comparison to untreated controls, with MIC_90_ values ≥ 90% inhibition.

### 2.5 Mono-species biofilm formation and treatment

Mono-species *G. vaginalis* biofilms were prepared by adding 200 μL of 1 × 10^7^ CFU/mL in NYC III into flat bottomed 96-well microtiter plates. Plates were incubated for 24 h anaerobically at 37 °C, before medium was removed and 200 μL fresh NYC III was applied for 24 h. After incubations, 200 μL of the desired antibiotic concentration (8–512 μg/mL for metronidazole, 0.0625–4 μg/mL for clindamycin) was applied wells and biofilms were incubated for a further 24-h anaerobically at 37 °C. Each plate contained untreated biofilms and negative controls (NYC III broth) to ensure sterility of culture medium. After treatment, colony forming units (CFU/mL) analysis was performed on mono-species *G. vaginalis* biofilms. For this, antibiotics were removed and biofilms were gently washed twice with sterile PBS. After washing, 100 μL of PBS was applied to wells and biofilms removed by scraping. Colony counting was performed using the Miles and Misra technique [24], in which serially diluted biofilms were applied in triplicate (20 μL) to Columbia blood agar plates. Plates were incubated for 48 h anaerobically, and CFU/mL estimation was performed by using the average of triplicate values.

### 2.6 Multi-species biofilm formation

**Fig. 1:**
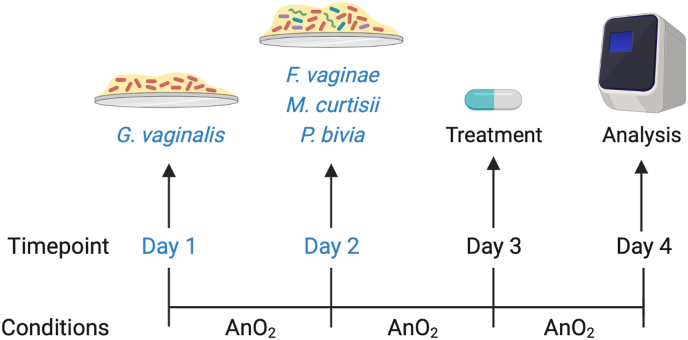
Timeline for growth of polymicrobial biofilm formation *in vitro*, depicting incubation times and conditions. AnO_2_ = anaerobic incubation. Biofilms were grown using a ‘*G. vaginalis* primed’ system, whereby this organism is allowed to colonise for 24 h, followed by additional BV-associated anaerobes (*F. vaginae, M. curtisii, P. bivia*). The four species are incubated together for a further 24 h before treatment is applied. Figure created using BioRender.

A four species BV-associated biofilm model was grown using a ‘*G. vaginalis* primed system’, whereby this organism is allowed to colonise for 24 h, followed by addition of remaining bacteria (Fig. 1). This is similar to previously developed biofilm models in which *G. vaginalis* was initially cultured in isolation, followed by additional organisms [[18], [25], [26]]. In brief, *G. vaginalis* was standardised to 1 × 10^8^ CFU/mL, and diluted 1:10 in NYC III broth to 1 × 10^7^ CFU/mL. From here, 500 μL was applied to wells of a 24-well microtiter plate containing sterile 13 mm Nunc™ Thermanox™ coverslips (Fisher Scientific, Loughborough, UK). Microtiter plates were incubated for 24 h under anaerobic conditions at 37 °C. After incubation medium was removed and *F. vaginae, P. bivia* and *M. curtisii* were standardised separately to 1 × 10^8^ CFU/mL. Each species was then combined to create a cocktail containing 1 × 10^7^ CFU/mL final concentration of each bacteria. Plates were incubated for a further 24 h under anaerobic conditions at 37 °C. On day 3, treatments were applied as described below.

### 2.7 Polymicrobial biofilm treatment

For polymicrobial biofilms, antibiotics were diluted in NYC III medium to desired concentrations (8 and 64 μg/mL metronidazole, 0.0625 and 0.5 μg/mL clindamycin). From here, 500 μL of each antibiotic was added to biofilms, alongside untreated (medium) controls. A similar protocol was followed for the CCB7.1 endolysin candidate, which was diluted to desired concentrations in buffer 1 to 128, 256 and 512 μg/mL, and added to biofilms alongside buffer 1 controls. For all treatments, plates were incubated for a further 24 h under anaerobic conditions at 37 °C.

### 2.8 DNA extraction and PMAxx treatment

Following multispecies biofilm treatment, coverslips were gently washed three times with sterile PBS and placed into bijou bottles containing 1 mL PBS. To remove bacteria from coverslips, the bijou bottles were sonicated at 35 kHz for 10 min, after which the sample was split into two 500 μL aliquots for live/dead quantification. One of the samples (live cells) was treated with PMAxx dye (Biotium, California, USA) as per manufacturer's instructions, with the other aliquot (total cells) not receiving PMAxx. After addition, treated samples were gently flicked to mix PMAxx dye and incubated in the dark for 10 min at room temperature as previously described for *in vitro* biofilms [22,27,28]. All samples were then exposed to the PMA-lite device (Biotium) for 15 min. After PMAxx treatment, DNA was extracted from all samples using the DNeasy kit (QIAGEN, Manchester, UK) as per manufacturer's instructions.

### 2.9 Quantitative PCR (qPCR)

All qPCR reactions were performed on an Applied Biosystems ViiA 7 real-time PCR system using 20 μL reactions. Each reaction mixture consisted of 10 μL 2x PowerUp SYBR green mastermix (Fisher Scientific), 7 μL Hyclone molecular grade water, 1 μL forward primer (10 μM), 1 μL reverse primer (10 μM) and 1 μL of DNA. This resulted in final primer concentrations of 0.5 μM per reaction. After preparation, plates were run with the following cycling conditions on the ViiA 7 analyser; 50 °C for 2 min, 95 °C for 2 min, 40 cycles of 95 °C for 3 s followed by 60 °C for 30 s. Primer sequences used throughout this study targeting each species or genus are highlighted in Table 1. For quantification of each organism, a standard curve was prepared using DNA extracted from known concentrations of the target bacteria, with colony forming equivalents per mL (CFE/mL) calculated as previously described [29].

**Table 1:** Primer sequences used during this study for qPCR to determine the composition of biofilm samples. Original references are highlighted.

Species	Forward primer (5′ to 3′)	Reverse primer (5′ to 3′)	Amplicon size (bp)	Reference
*Gardnerella vaginalis*	GGAAACGGGTGGTAATGCTGG	CGAAGCCTAGGTGGGCCATT	125	[30]
*Fannyhessea vaginae*	GTTAGGTCAGGAGTTAAATCTG	TCATGGCCCAGAAGACC	157	
*Mobiluncus curtisii*	GCGATGGTTCCAGAGATGGGCCAGCCTT	CACGAGTCCCCGGCCGAA	148	
*Prevotella* species	GGGATGCGTCTGATTAGCTTGTT	CTGCACGCTACTTGGCTGGTTC	179	

### 2.10 Scanning electron microscopy

To assess the ultrastructure of polymicrobial biofilms formed on coverslips, scanning electron microscopy (SEM) was used as previously described [27,31]. Briefly, biofilms were fixed using 2% paraformaldehyde, 2% glutaraldehyde, 0.15% alician blue power and 0.15 M sodium cacodylate. This was followed by counterstaining with uranyl acetate and gradient dehydration in ethanol (30–100%). Samples were then sputter-coated using gold/palladium and visualised using a JEOL JSM-6400 SEM machine at 1000x and 5000x magnification (JEOL Ltd).

### 2.11 Data analysis

Data was analysed and graphs compiled using a combination of Microsoft Excel (version 16.60) and GraphPad PRISM (version 9). Normality tests were performed and appropriate statistical analysis performed. For example, non-parametric data was analysed using Mann-Whitney *U* test or Kruskall-Wallis with Dunn's post-hoc where appropriate. Statistical significance was defined as p < 0.05 throughout.

## 3 Results

### 3.1 Antibiotic treatment of monospecies *G. vaginalis* biofilms

**Fig. 2:**
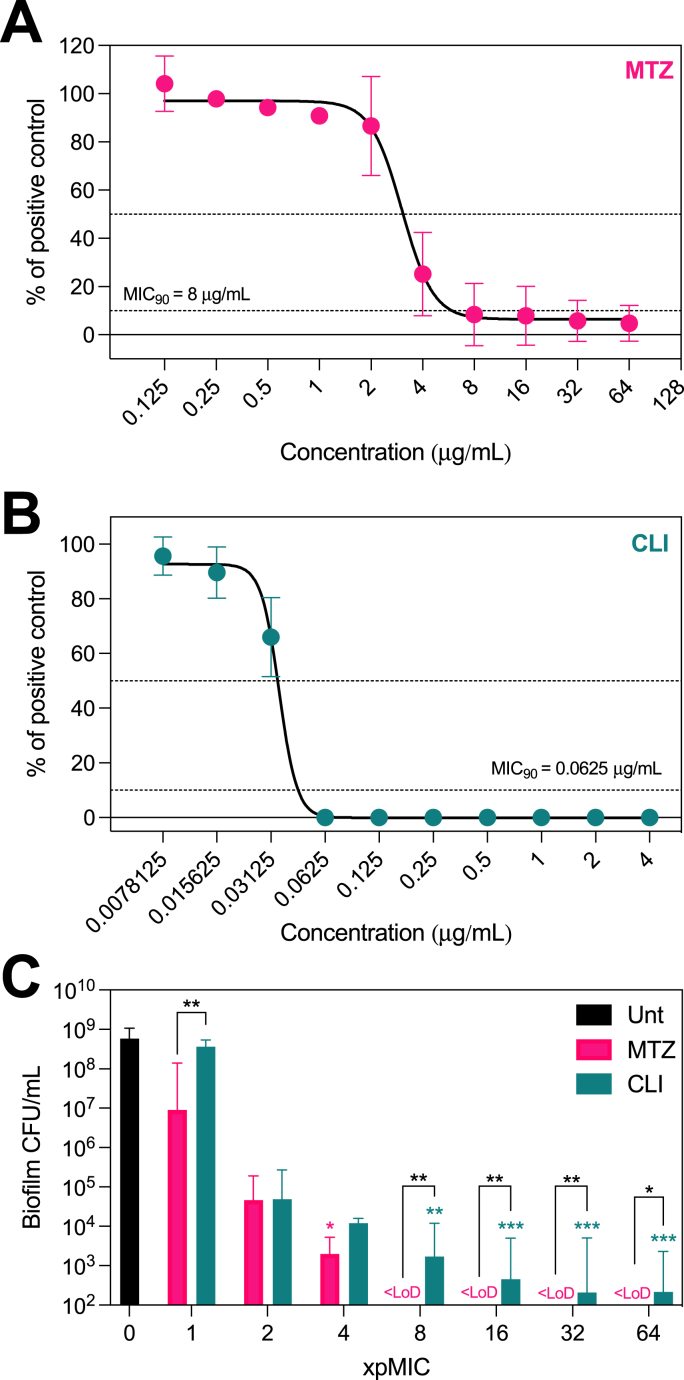
Characterising the efficacy of antibiotics against *G. vaginalis.* Displaying the planktonic inhibition of *G. vaginalis* ATCC 14018 following metronidazole (A) and clindamycin (B) treatment, represented as a percentage growth in relation to untreated [positive] controls. Each antibiotic was additionally screened against mono-species *G. vaginalis* biofilms (C), and viability presented as CFU/mL. Graphs display mean ± standard deviation (A,B) or median ± 95% CI (C). Statistics represent Kruskall-Wallis with Dunn's post-hoc, where Asterix above bars indicates a significant difference with the untreated control. Connecting lines represents direct comparison between clindamycin and metronidazole performed using Mann-Whitney tests adjusted using the Holm-Sidak method where *p < 0.05 and **p < 0.01 and ***p < 0.001. MTZ; metronidazole, CLI; clindamycin, LoD; limit of detection. All experiments are n = 3.

As mentioned, frontline therapy for BV remains largely limited to two antibiotics. A common pitfall of these treatments is the large discrepancy between bacterial susceptibility in a planktonic and biofilm-embedded state. To understand this in the context of BV, we characterised the efficacy of clindamycin and metronidazole against *G. vaginalis* ATCC 14018. As expected, this strain was susceptible to both antibiotics when assessed planktonically. The pMIC concentrations were 8 μg/mL (Fig. 2A, resistance breakpoint ≥32 μg/mL) and 0.0625 μg/mL (Fig. 2B, resistance breakpoint ≥8 μg/mL) for metronidazole and clindamycin respectively, which is in line with previous publications [[14], [32]].

Extending this to biofilm-embedded *G. vaginalis*, we screened the efficacy of each antibiotic at concentrations ranging from 1 to 64X pMIC (8–512 μg/mL for metronidazole, 0.0625–4 μg/mL for clindamycin). Untreated *G. vaginalis* biofilms yielded 1 × 10^8^ – 1 × 10^9^ CFU/mL after 48-h incubation (Fig. 2C). Although the pMIC inhibited planktonic growth of this isolate, 9 × 10^6^ CFU/mL and 3.6 × 10^8^ CFU/mL were recovered from biofilms treated with 1X pMIC metronidazole and clindamycin respectively (adjusted p = 0.006 comparing antibiotics, Mann-Whitney test). With increasing concentrations, a dose-dependent reduction in biofilm viability was observed following each antibiotic. For each compound, detectable G. vaginalis was recoverable up to 8X pMIC (metronidazole) and >64X pMIC (clindamycin), reiterating the resilience of biofilm-embedded *G. vaginalis* against antibiotic treatment.

### 3.2 Polymicrobial BV biofilm development

Based on current understanding, it is proposed that *G. vaginalis* may be the primary coloniser of BV associated biofilms [33,34], followed by attachment of additional BV-associated anaerobes. To recapitulate this *in vitro*, we thus sought to explore a '*G. vaginalis* primed'biofilm model, which has been previously used in dual and more recently tri-species biofilm models of BV [20,25]. In this system *G. vaginalis* was initially incubated on coverslips for 24-h, followed by addition of *F. vaginae, M. curtisii* and *P. bivia* for 24-h.

**Fig. 3:**
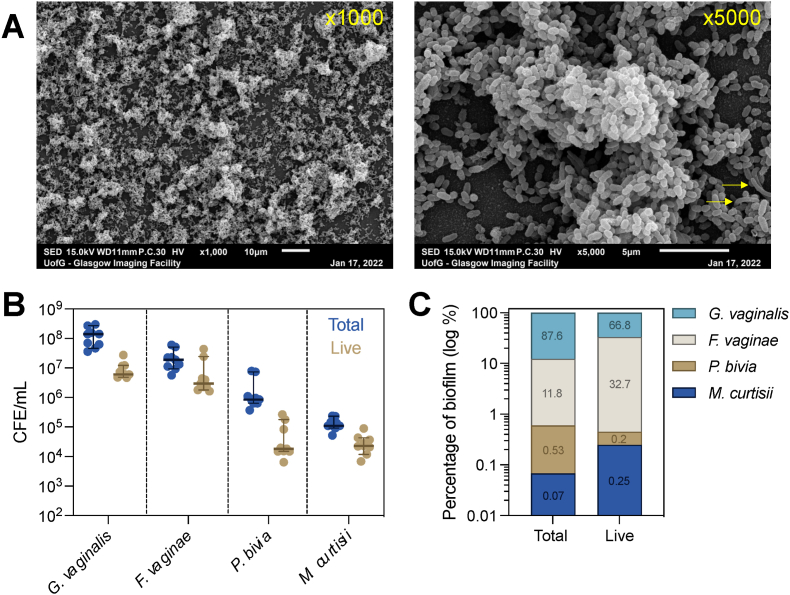
Summary composition of polymicrobial biofilms. Scanning electron microscopy of biofilms at 1000x and 5000x magnification (A). Yellow arrows indicate morphologically distinct, longer and curved bacilli which putatively represent *M. curtisii*. Displaying the CFE/mL values of total and live quantities of each organism in biofilm samples as determined by live/dead qPCR (B, n = 3). Graphs display median ±95% confidence interval. The percentage composition of biofilms (C). The y-axis is log_10_ scaled, with exact percentage values displayed on each bar. (For interpretation of the references to colour in this figure legend, the reader is referred to the Web version of this article.)

Following biofilm development, the ultrastructure of polymicrobial biofilms was investigated using SEM at 1000x and 5000x magnification (Fig. 3A). As expected, this imaging revealed the formation of a dense, three-dimensional biofilm, characterised by large clusters of interlinked bacteria. Using live/dead qPCR, we confirmed that all input species colonised the resulting biofilm (Fig. 3B), where *G. vaginalis* was the most abundant (87.6% of total cells, 66.8% of live cells, Fig. 3C), followed by *F. vaginae* (11.8% of total cells, 32.7% of live cells). In contrast, both *P. bivia* and *M. curtisii* were less abundant with roughly 1 × 10^4^ CFE/mL live cells, comparable to previously published dual-species models whereby *G. vaginalis* dominates [[20], [26]]. These data are expected given that a *G. vaginalis* initiated system was utilised in this study. Nonetheless, recoverable levels of each organism were detectable in polymicrobial biofilms.

### 3.3 Antibiotic treatment of polymicrobial BV biofilms

**Fig. 4:**
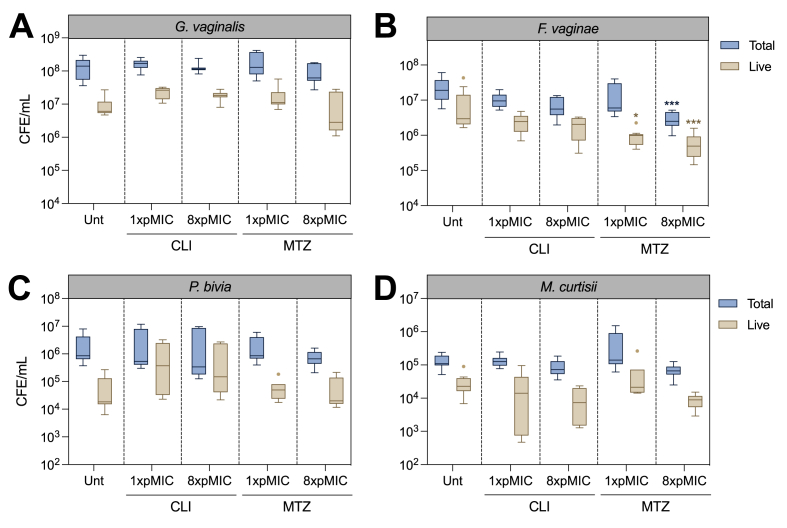
Polymicrobial biofilm treatment with antibiotics. Metronidazole and clindamycin were applied at 1X and 8X pMIC of *G. vaginalis*. Graphs display Tukey boxplots of total and live *G. vaginalis* (A), *F. vaginae* (B), *M. curtisii* (C) and *P. bivia* (D), where horizontal lines represent the median and potential outliers displayed as single points. Statistics are Kruskall-Wallis test with Dunn's post-hoc, performed separately for total and live cells. ***p < 0.001. Unt = Untreated controls. All experiments are n = 3.

To investigate how this polymicrobial model responded to antibiotic therapy, both metronidazole and clindamycin were applied at 1x and 8X pMIC of *G. vaginalis.* These concentrations were selected based on monospecies biofilm experiments - where minimal alterations were observed following 1X pMIC, with more pronounced reductions in viability at 8X pMIC of each antibiotic. Treatment was applied to polymicrobial biofilms for 24-h, and the viability of each species was assessed using live/dead qPCR (Fig. 4).

Following antibiotic treatment, there were no significant alterations to the total number of *G. vaginalis* cells compared with untreated controls (Fig. 4A). No reduction was also observed when assessing live *G. vaginalis* cells; this contrasted with mono-species biofilm treatment, where live cell count was significantly impacted by the antibiotics. Despite a subtle reduction in the median live *G. vaginalis* quantity at 8X pMIC metronidazole, this did not reach statistical significance in comparison to untreated biofilms (adjusted p > 0.05). These findings were consistent for both *P. bivia* (Fig. 4C) and *M. curtisii* (Fig. 4D) – where no significant alterations in total or live cells were found (adjusted p > 0.05). The only significant difference was a reduction in live *F. vaginae* at 1X and 8X pMIC metronidazole (adjusted p = 0.01 and 0.0002 respectively), the latter of which also reduced the total *F. vaginae* load (adjusted p < 0.001, Fig. 4B). Conversely, no significant alterations in total or live *F. vaginae* were found following clindamycin therapy.

### 3.4 Endolysin treatment of polymicrobial BV biofilms

Data from our polymicrobial model showed no, or limited, alterations in biofilm viability following antibiotic therapy. As such, we sought to evaluate the efficacy of engineered phage endolysins which were developed to target *Gardnerella* spp., and represent a promising novel therapy in combatting BV-associated biofilm communities.

**Fig. 5:**
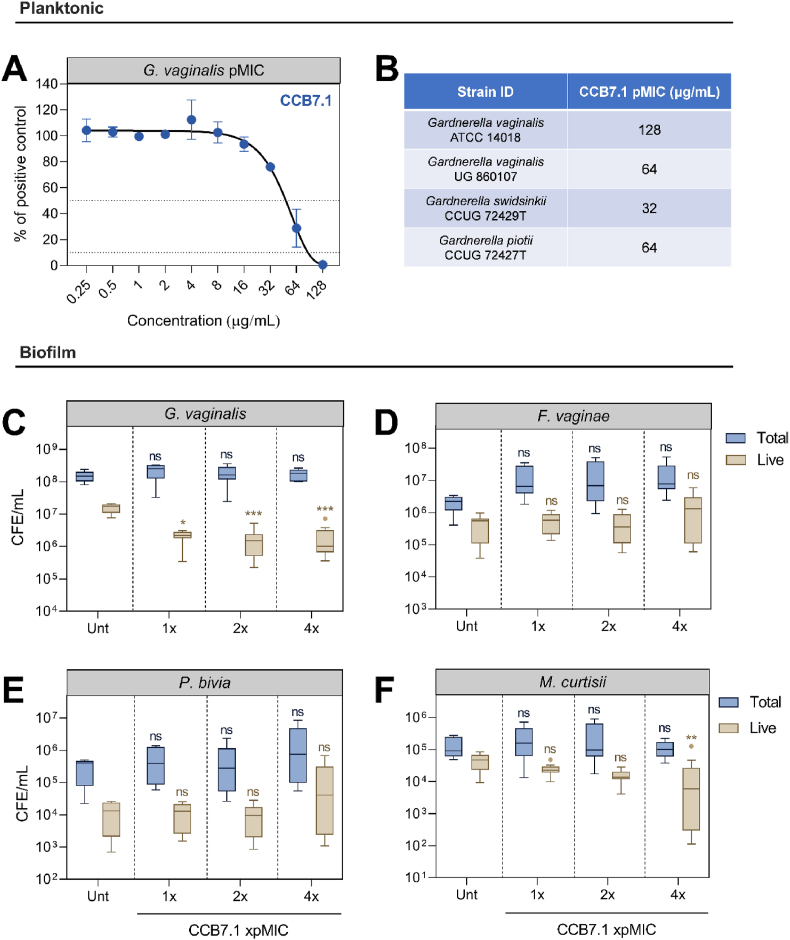
Testing a *Gardnerella* endolysin against polymicrobial BV biofilms. Figure display the pMIC of CCB7.1 against *G. vaginalis* ATCC 14018 (A), with additional *Gardnerella* species pMICs (B). The CCB7.1 endolysin was screened against polymicrobial biofilms at 1–4X pMIC, with figures displaying the total and live *G. vaginalis* (C), *F. vaginae* (D), *P. bivia* (E) and *M. curtisii* (F) after treatment. Graphs display mean ± standard deviation (A) or Tukey boxplots (B–E). Statistics are Kruskall-Wallis test with Dunn's post-hoc, performed separately for total and live cells, where ns means no significant difference, *p < 0.05, **p < 0.01 and ***p < 0.001. Unt = untreated controls, n = 3 for all experiments excluding B, where n = 2.

Although previous studies have documented success using these antibacterial proteins *in vitro*, we aimed to build upon this data and trial one candidate (CCB7.1) against our more complex system. As expected, this protein was effective against the *G. vaginalis* ATCC 14018 when assessed planktonically. The pMIC value against this isolate was 128 μg/mL (Fig. 5A), corresponding with previously published values [12]. Furthermore, we also observed efficacy against additional *Gardnerella* isolates such as an additional *G. vaginalis* strain (64 μg/mL), *G. swidsinskii* (32 μg/mL) and *G. piotii* (64 μg/mL) (Fig. 5B).

The endolysin candidate was next applied to pre-formed polymicrobial biofilms, to investigate its efficacy against biofilm-embedded *G. vaginalis* alongside additional BV-associated anaerobes. Three concentrations of endolysin were applied, representing 1X, 2X and 4X pMIC (128, 256 and 512 μg/mL). After addition, we observed a significant reduction in the viable levels of *G. vaginalis* using live/dead qPCR (adjusted p < 0.05 for all concentrations, Fig. 5C). This represented a 1–2 log_10_ reduction in viable *G. vaginalis* at all tested concentrations, compared with untreated controls. Although reduced, it is noteworthy to mention that *G. vaginalis* remained viable at ≈1 × 10^6^ CFE/mL. Promisingly, the reduction was consistent, and more pronounced than observed for the current standard of care antibiotics against *G. vaginalis*. Assessment of additional BV-associated anaerobes demonstrated a reduction in live *M. curtisii* at the highest tested concentration (adjusted p = 0.007), although this species was the least abundant in the model system. Such a reduction may be due to biofilm destabilisation from reductions in *G. vaginalis,* rather than direct bacteriolytic activity. Endolysin treatment did not impact the total level of any species, which notably included *G. vaginalis*.

**Fig. 6:**
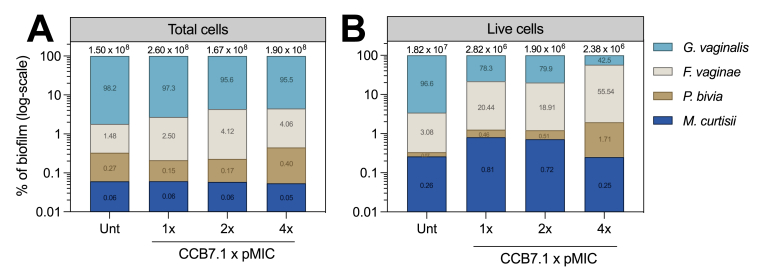
Compositional shifts following endolysin therapy. Graphs display the percentage abundance of total (A) and live (B) cells following addition of the CCB7.1 endolysin at increasing concentrations. The total and live cell numbers are displayed above each bar, and y-axes are log_10_ scaled. Exact percentage values are displayed on bars corresponding to each species. Unt = Untreated controls. All experiments are n = 3.

Endolysin treatment did not alter total cell counts within the biofilm, although the live bacterial load reduced ≈1-log_10_, which was almost entirely due to *G. vaginalis* (Fig. 6A and B). From a compositional perspective, *G. vaginalis* equated to >95% of live cells in untreated controls, which reduced to 78.3%, 79.9% and 42.5% with increasing doses of CCB7.1. Notably, a concomitant increase in the percentage abundance of live *F. vaginae* (Fig. 6B) was observed in the model alongside the reduction in *G. vaginalis* abundance*.* Although *F. vaginae* did not significantly shift in comparison to untreated controls, its lack of reduction equated to a larger proportion of the live bacterial load when *G. vaginalis* was reduced.

## 4 Discussion

Metronidazole and clindamycin are first-line antibiotic therapies for BV*,* applied topically or given orally. Whilst these antibiotics provide good coverage of BV-associated anaerobes [35], such therapies are disappointing and appear insufficient for many patients with evidence demonstrating a recurrence rate of >50% [10,[35], [36], [37]]. This is not only hugely debilitating for patients who suffer from recurrent BV, but also highlights a distinct lack of alternative therapies available. One reason believed to drive these high rates of recurrence is the formation and recalcitrance of BV-associated biofilms which are inadequately eradicated by existing antibiotic therapy [4]. It is therefore essential that existing and novel therapeutics targeting BV-associated bacteria are assessed using *in vitro* models which are more reflective of this biofilm-based clinical presentation.

Accordingly, in this study we first sought to develop a polymicrobial biofilm-based model which is more replicative of clinical dysbiosis, and harness this model to investigate the impact of polymicrobial interactions on antimicrobial tolerance in BV. As expected, mono-species *G. vaginalis* biofilms were more tolerant of antibiotics (metronidazole, clindamycin) compared with planktonic cultures. Interestingly, our *G. vaginalis*-containing, four-species model showed almost complete resilience to current antibiotics prescribed for BV (metronidazole, clindamycin), with no statistical change in the composition of *G. vaginalis* cells even at the highest concentrations tested. Thus, whilst *G. vaginalis* mono-species biofilms serve as a useful preliminary screening tool, it is clear that this is not the only bacterium involved in BV-associated biofilms – and the presence of additional species such as *F. vaginae*, *P. bivia* and *M. curtisii* may alter biofilm susceptibility to antimicrobial challenge. In this regard, recent work has demonstrated that polymicrobial interactions *in vitro* facilitate increased resistance to metronidazole and clindamycin [32], supporting these results and providing further evidence for the involvement of recalcitrant polymicrobial biofilms in BV treatment failure.

It is also worth considering the molecular approach used to quantify polymicrobial biofilms in the current study may further increase viability estimates in comparison with colony counting. Specifically, the ability of live/dead qPCR to detect viable but non-culturable cells (VBNCs) and persister cells within biofilms, which would not be detectable using conventional colony counting culture methodology routinely used for mono-species analysis. As such, colony counting more accurately reflects the presence of culturable cells within biofilms, rather than viable cells (including both culturable and non-culturable cells). This has been previously suggested in the context of wound biofilm models comparing colony counting and qPCR [38], and it is likely that this would extend to BV-associated biofilms used in this study.

Following antibiotic treatment, we then sought to test the impact of one novel BV therapy, a bacteriophage-encoded endolysin, upon our model. Given the limited therapeutic options for BV treatment, there has been recent interest in the use of endolysin proteins as a novel alternate therapeutic. These proteins are naturally encoded within bacteriophage genomes, and offer highly selective lysis of target bacteria when recombinantly produced. Previous research has documented promising results following endolysin treatment of biofilm-embedded *G. vaginalis* [12,14,18]. Herein, we focused on one of these aforementioned endolysin candidates, CCB7.1, which was selected based on its previously elucidated ability to prevent *G. vaginalis* biofilm formation and disrupt pre-formed *G. vaginalis* biofilms [12]. Importantly, and as expected, CCB7.1 was previously shown to be ineffective against commensal lactobacilli [12] – offering selective therapy of *Gardnerella* spp. without off-target impacts on beneficial flora.

After first demonstrating that CCB7.1 was effective against additional, and more clinically representative *Gardnerella* isolates (*G. vaginalis, G. swidsinskii, G. piotii*), we evaluated the impact of this targeted *Gardnerella* endolysin against a more complex, BV-representative consortium using our polyspecies BV model. Our data demonstrate a significant reduction in the viability of *G. vaginalis* within this model following treatment with the engineered phage endolysin CCB7.1 at three concentrations (128, 256 and 512 μg/mL), representing a 1–2 log_10_ reduction in viability compared with untreated controls. Thus, whilst the antimicrobial activity of CCB7.1 is encouragingly maintained when *G. vaginalis* is embedded in a more diverse *in vitro* biofilm, the magnitude of impact was less than observed in mono- and dual-species biofilm systems [12].

While it was generally expected that the efficacy of endolysin treatment would reduce in a multi-species model compared with monoculture systems, this relative reduction in efficacy compared favourably to the incumbent standard of care antibiotics metronidazole and clindamycin. One reason for reduced endolysin efficacy may result from the formation of a *G. vaginalis* primed biofilm model used in this study. Specifically, priming biofilms with *G. vaginalis* will likely result in partial coverage by secondary bacteria (*F. vaginae, M. curtisii, P. bivia*) which prevent immediate contact between lysins and *G. vaginalis*. This more accurately mimics the proposed model of polymicrobial biofilm formation in BV where *G. vaginalis* forms the initial biofilm scaffold [8,25], and it is thus reassuring that CCB7.1 remained capable of reducing *G. vaginalis* viability when grown in this system.

Taken together, the data obtained in this study underline the importance of using polyspecies models for *in vitro* evaluation of emerging therapeutic candidates. It is important that more clinically representative data outputs are obtained at earlier stages of preclinical development thereby reducing attrition rates later in the clinical development cycle. Indeed, some researchers have recently started to transition beyond simple BV biofilm models [[20], [32]], and this work, in combination with the model developed in this current study, will form a useful basis for more accurate screening of BV therapies moving forward.

Although the evidence to date suggests that endolysin therapy is promising for BV, such a targeted approach is not devoid of disadvantages and an important consideration moving forward will be the implications of selective knockdown of this organism *in vivo*. For example, it is known that BV associated biofilms are polymicrobial in nature and knockdown of *Gardnerella* spp. spp. may result in enrichment of non-target species. This may be of particular relevance given that *F. vaginae* was recently reported to display a larger inflammatory burden on a 3D human cervix model when compared with *G. vaginalis* [39]. This finding may lend itself to the use of endolysin cocktails which can target several BV-associated anaerobes, in a similar fashion to the approach used in bacteriophage therapy [40]. However the development of endolysins against *P. bivia* and other Gram-negative bacteria represents a continual challenge in the field [41].

A limitation of this study is that only a single method (live/dead qPCR) was used to characterise polymicrobial biofilm viability and composition. Although the propoidium monoazide based assay is widely reported for *in vitro* polymicrobial biofilms [22,28,[42], [43], [44]], the use of additional techniques may provide a greater insight into the biofilm structure and bacterial organisation following endolysin therapy. In this regard, one technique which could be utilised to further characterise these biofilms is fluorescence *in situ* hybridisation using peptide nucleic acid probes (PNA FISH). This technique offers the benefit of complimentary imaging and quantitative assessment of biofilms, and PNA FISH probes have been developed and optimised for both *G. vaginalis* and *F. vaginae* [[20], [45], [46]]. Ensuing research may seek to use a combination of approaches for biofilm characterisation to provide simultaneous assessment of species-specific viability, biofilm structure and composition.

In conclusion, this study outlines the development of a novel polymicrobial *in vitro* biofilm system which is more replicative of clinical BV. The bioactivity of metronidazole, clindamycin and CCB7.1 were then assessed against this more complex model. Our data demonstrate tolerance of this model against standard of care antibiotic therapy, and a reduction in *G. vaginalis* viability following endolysin application. Although significant, these viability impacts following endolysin administration were less pronounced than previously observed against mono-species biofilms, which reiterates the importance of using more diverse and clinically relevant biofilms for screening therapies in BV. Whilst the microbiota in BV extends well beyond the four species used in this study [3], this model does represent an advancement on ubiquitously used mono-species *G. vaginalis* biofilms. Moving forward, future studies may seek to trial endolysins in cocktails or evaluate synergy with existing antibiotics to provide a larger spectrum against the polymicrobial biofilm model. Additionally, it is important that the collective findings documenting the efficacy of *Gardnerella-*targeted endolysins are translated into clinically meaningful outputs – such as a reduction in the inflammatory potential of biofilms following endolysin treatment.

## Funding

The current study was supported by the 10.13039/501100015044National Biofilms Innovation Centre (NBIC) under project number: 04POC21-235 and an Innovation Seed Funding award from Scottish Universities Life Sciences Alliance.

[[26], [32], [46], [47]].

## CRediT authorship contribution statement

**William Johnston:** Methodology, Investigation, Formal analysis, Visualization, Writing – original draft, Writing – review & editing. **Alicia Ware:** Investigation, Formal analysis, Writing – review & editing. **Willemijn Frederique Kuiters:** Investigation, Formal analysis, Visualization. **Christopher Delaney:** Methodology, Software, Writing – review & editing. **Jason Lee Brown:** Methodology, Formal analysis, Writing – review & editing. **Suzanne Hagen:** Conceptualization, Writing – review & editing, Supervision, Project administration, Funding acquisition. **David Corcoran:** Conceptualization, Investigation, Resources, Writing – original draft, Project administration, Funding acquisition. **Matthew Cummings:** Conceptualization, Investigation, Resources, Writing – original draft, Project administration, Funding acquisition. **Gordon Ramage:** Conceptualization, Methodology, Writing – original draft, Project administration, Funding acquisition. **Ryan Kean:** Conceptualization, Methodology, Investigation, Writing – original draft, Writing – review & editing, Visualization, Supervision, Project administration, Funding acquisition.

## Declaration of competing interest

The authors declare the following financial interests/personal relationships which may be considered as potential competing interests: Matthew Cummings and David Corcorcan of CC Biotech Ltd have a patent for the clinical use of the endolysin(s) described in this study.

## Data Availability

Data will be made available on request.
